# Exercise and diabetes: relevance and causes for response variability

**DOI:** 10.1007/s12020-015-0792-6

**Published:** 2015-12-07

**Authors:** Anja Böhm, Cora Weigert, Harald Staiger, Hans-Ulrich Häring

**Affiliations:** Department of Internal Medicine IV, Division of Endocrinology, Diabetology, Angiology, Nephrology, and Clinical Chemistry, University Hospital Tübingen, Eberhard Karls University Tübingen, 72076 Tübingen, Germany; Institute for Diabetes Research and Metabolic Diseases of the Helmholtz Center Munich at the Eberhard Karls University Tübingen, Tübingen, Germany; German Center for Diabetes Research (DZD), 85764 München-Neuherberg, Germany

**Keywords:** Non-response, Adverse response to exercise, Lifestyle intervention, Exercise resistance, Insulin sensitivity, Glucose homeostasis, Glucose tolerance

## Abstract

Exercise as a key prevention strategy for diabetes and obesity is commonly accepted and recommended throughout the world. Unfortunately, not all individuals profit to the same extent, some exhibit exercise resistance. This phenomenon of non-response to exercise is found for several endpoints, including glucose tolerance and insulin sensitivity. Since these non-responders are of notable quantity, there is the need to understand the underlying mechanisms and to identify predictors of response. This displays the basis to develop personalized training intervention regimes. In this review, we summarize the current knowledge on response variability, with focus on human studies and improvement of glucose homeostasis as outcome.

## Introduction

The global epidemic of type 2 diabetes burdens humankind. The WHO projects that diabetes will be the 7th leading cause of death in 2030. For prevention, healthy diet as well as achievement and maintenance of normal body weight are recommended. Furthermore, at least 30 min of regular, moderate-intense physical activity five times a week is required [1, 2]. Nevertheless, our strategies to prevent type 2 diabetes are still insufficient; since decades, a major purpose of research is to develop reasonable prevention strategies and to specify detailed pathomechanisms leading to diabetes.

There are myriads of intervention studies dealing with the best exercise type, frequency, intensity, and duration, further sophisticated by additional diets [3–20 and many more], and the scientific discussion is still ongoing. Indeed, positive effects of regularly performed exercise on cardiorespiratory fitness and metabolic control are without dispute. In most of the well-known diabetes prevention studies as DPS, DDP, HERITAGE, LookAHEAD, STRRIDE, Da Qing Diabetes Study, TULIP, and others, the risk reduction for diabetes, the metabolic syndrome or cardiovascular events ranges around 35 % [4, 21–35]. Despite this knowledge, less than 40 % of European countries developed national recommendations for physical activity [36].

## Response variability

Most of the conducted studies found not only improvements in metabolic and cardiorespiratory endpoints after training intervention, but also highly variable inter-individual responses [37–39]. Maximum oxygen uptake (VO_2_max) is the standard parameter of cardiorespiratory fitness and is widely used to document the effectiveness of training. The HERITAGE trial identified low responders and high responders for improvements of VO_2_max [40]. A similar variability for glucose homeostasis, reflected by insulin sensitivity, acute insulin response, glucose effectiveness, and glucose disappearance index was shown [41]. The general distribution of individual changes seems to have a two-sided shape, ranging from high responders to even adverse responders that show a deterioration of the respective endpoint. Notably, the term “non-response to exercise” always needs a clear association with a specific endpoint. It is used with respect to changes in several, different parameters assessed before and after training, e.g., fitness, cardiovascular events, muscle mass, metabolic risk profiles, lipid metabolism, insulin resistance, glucose tolerance, and others. In this review, we focus on the failure to improve whole-body glucose homeostasis after training interventions in humans. Physical activity is often included in lifestyle intervention programs combining dietary regimes with exercise, and sometimes we also refer to data based on lifestyle intervention. Since it is not possible to differentiate between exercise-dependent and exercise-independent effects in these studies, this is always clearly stated.

What about the quantity of these non-, low-, or even adverse responders? As recently reviewed [42], the number of adverse responders with respect to fasting insulin including six exercise training studies (HERITAGE, DREW, INFLAME, STRRIDE, MARYLAND, and JYVASKYLA) averaged 8.3 %. Non-response defined as no improvement regarding glucose homeostasis, leads to 7–63 % non-responders [41, 43–49]. For further details, see Table 1. Most of the conducted studies are performed without a control group. Thus, the opinion exists that exercise might cause adverse metabolic effects for some individuals. However, a study performed with 87 participants including a control group [45], demonstrated clearly a decreased number of an adverse response (41 %) versus 76 % in control group; the adverse response was defined as increased fasting glucose, 2-h glucose, and triglycerides, as well as a decrease for HDL-cholesterol.

**Table 1:** Quantity of non-responders

Citation	Population	Intervention	Duration	Outcome	Non-responders (%)^a^
Boulé [41]	*n* = 596, healthy	Endurance training, 3×/week, 55–75 % VO_2_max,	20 weeks	Insulin sensitivity	42
Borel [46]	*n* = 104, abdominally obese/dyslipidemic	160 min/week moderate-intensity exercise and −500 kcal per day, pedometer use	12 months	Glucose tolerance status	62.5
Hagberg [49]	*n* = 110, healthy	endurance training, 3×/week, 50–70 % VO_2_max	26 weeks	Insulin sensitivity	25
Yates [45]	*n* = 29, prediabetic	education program with pedometer use	12 months	2-h glucose	7^b^
Winett [44]	*n* = 159, prediabetic	Resistance training, 2 ×/week	3 months	2-h OGTT	44^c^
Stephens [48]	*n* = 42, diabetic	Aerobic, resistance training, or combination thereof	9 months	Combination of HbA1c, % body fat, BMI, muscle mitochondrial content	21
Osler [47]	*n* = 14, prediabetic	Nordic walking, 5 h/week, unsupervised	20 weeks	Glucose tolerance status	36

Quantity of non-responders with respect to glucose homeostasis

^a^Meaning no improvement, unless stated otherwise

^b^Adverse response

^c^Estimated from graph

Notably, the failure to improve one metabolic factor is not necessarily reflected by a non-response in other variables, e.g., VO_2_max, and vice versa [50]. Although there is a clear positive correlation of VO_2_max and insulin sensitivity in the general population [51–53] and an increase in VO_2_max correlates with the improvement in glucose homeostasis [54] and insulin sensitivity [55] in large lifestyle intervention programs, this is not true for each individual. In 202 diabetic individuals of the HART-D study, only 37 % had a marked increase in VO_2_max, but all profited regarding metabolic parameters, irrespective of VO_2_max response [56]. Furthermore, metabolic fitness parameters like respiratory exchange ratio, maximal heart rate, and maximal ventilatory equivalent do not relate to changes in aerobic capacity [57].

Thus, despite a relevant exercise-related improvement of systolic blood pressure, body weight, VO_2_max, lipid profile, etc., one may not have a beneficial effect on glucose homeostasis; this adds even more complexity to this issue.

It is still under debate [11, 16, 42, 43, 58–62], which training intervention is the best, and this will not be in focus of this review. However, a recent study gave hint for a combination of low-amount/vigorous-intensity aerobic exercise and resistance training being favored [63]. High-intensity interval training has been practiced by athletes for some time [64]; recently it receives much interest as promising part of lifestyle intervention programs [65]. It can be superior to moderate-intense, time-consuming continuous training in improving cardiorespiratory fitness [66], and beneficial effects on glucose homeostasis or insulin sensitivity have been shown after just short training duration [67, 68]. If high-intensity interval training will be advantageous, and which subpopulation is suitable to that, we will learn from future randomized, controlled studies. Additionally, the question arises, if the highly individual responses to exercise might be overcome by different training regimes.

To sum up, individual exercise response is known for several years now [11, 37, 57, 69, 70], but shifting the focus on non-response in terms of glucose homeostasis is just beginning [29, 43, 46–48, 56, 71, 72].

### Prediction of and mechanisms for failure

Understanding and defining the individual susceptibility for non-response will be a major purpose in the future. This is the basis for the development of personalized training strategies to prevent and treat type 2 diabetes. Regarding success-predictive baseline values, our knowledge is limited to few studies and endpoints, as reviewed by [73], and the results are partly complementary. Of course, personal adherence to lifestyle intervention is a major fundament for success [74]; thus, exercise studies should exclusively be supervised.

Beyond this, in the HERITAGE study, baseline values were found to account for ~40 % variability in training-related changes; but only for some traits, such as submaximal heart rate and blood pressure, where high baseline levels were associated with major exercise-driven improvements [37]; but not for baseline VO_2_max, HDL, age, nor for sex and race [39], where no relationships were found; contrarily, age was mentioned as a relevant variable in dose-responsiveness to exercise [75], as older adults might require higher doses of training. Another study showed, that there are no non-responders in elderly practicing a prolonged resistance training [60]. Notably, insulin sensitivity or glucose tolerance was not among the endpoints of this study [60]. Additionally, women with low fitness at baseline were shown to have greater exercise-related fitness improvements [76].

For glucose homeostasis, there are quite little data. Risk factors for non-response are speculated, but far from being comprehensively understood. But recognizing these individuals that fail to profit from exercise is of major importance. In a 9 months exercise study, long duration of type 2 diabetes and increases in serum free fatty acids (FFA) were positively associated with HbA1c changes, whereas serum adiponectin levels and muscle protein content of peroxisome proliferator-activated receptor γ coactivator 1α (PGC1α) correlated inversely with changes in HbA1c [77]. In plasma, reduction of ceramides was correlated with exercise-related improvements in insulin sensitivity [78]. A whole blood gene expression analysis after 12 weeks of lifestyle intervention in Latino adolescents showed up-regulated genes, e.g., for insulin signaling, glucose uptake, and glycogen storage as well as down-regulation of genes involved in inflammatory pathways, and the analysis exhibited five times the number of regulated transcripts in insulin sensitivity- responders compared to non-responders in terms of insulin sensitivity [79]. This might point to a reduced adaption to training stress in non-responders. From the Diabetes Prevention Program, we know that low insulin secretion and low insulin sensitivity at baseline generally predict higher diabetes risk regardless of the treatment regime [80]. Our own data from the TULIP study showed low insulin secretion and sensitivity, low cardiorespiratory fitness, high liver and visceral fat, as well as high fetuin A predictive for non-response regarding glucose homeostasis [55, 72, 81, 82], whereas age, sex, and BMI at baseline were not predictive. Notably, this was a lifestyle intervention study, and conclusions on exercise-specific changes can only be speculated. Indeed, exercise-driven improvement of glucose tolerance was only shown in insulin-resistant individuals with adequate insulin secretion [83].

Thus, there are several pathomechanisms conceivable leading to failed exercise-related improvement of glucose homeostasis: most expectedly, no improvement in insulin sensitivity with consequences in all insulin-responsive tissues (muscle, adipose tissue, liver, brain) leads to less glucose deposition and increased endogenous glucose production; furthermore, less insulin secretion or altered glucose/fatty acid metabolism could be pathogenic. Presumably, the non-response regarding glucose homeostasis might be a combination of these factors, and distinguishing this will be very sophisticated.

Is worse glucose homeostasis per se objectively a risk factor for non-response? There is some evidence given by several exercises [45, 84–86] and lifestyle intervention studies [29, 46, 87] that individuals with higher metabolic burden seem to profit more. Contrarily, in another study, responders in terms of glucose tolerance had better glucose homeostasis values at the beginning than non-responders [47]; additionally, women at lower genetic risk for obesity (calculated by a risk score dependent on 21 SNPs associated with BMI variation) showed more favorable responses regarding resistance training-associated changes of body fat composition [88]. These partly conflicting results might be explained by a ceiling-effect for some variables, different populations and study settings. Alternatively, there might be a threshold in any metabolic parameter—perhaps insulin secretion?—beyond which the benefit suddenly converts to the opposite. Until now, there is too much speculation on pathomechanisms and we clearly require further studies in well-defined populations under controlled conditions for better characterization of responders and non-responders.

### Genetic aspects of non-response

Already in the 1980s, the relevance of heredity in exercise-induced adaptations was shown [89]. For exercise-related improvements of VO_2_max, the heritability is reported to be about 47 % [40, 90]. Single nucleotide polymorphisms (SNPs) are found to play a role in the training-induced changes in VO_2_max [91]; also for the endpoint muscle strength this was shown [92]. A combination of several SNPs contributes to ~50 % of the inter-individual variance in changes of VO_2_max [93, 94], pointing to a multifactorial inheritance of general non-response. A genetic variant in NDUFB6, encoding for complex I of the respiratory chain, can modify the individual response of the ATP synthase flux, even independently from exercise-related improvements of insulin sensitivity [95]. For metabolic syndrome in general, risk allele carriers of IL6R had more profit from a lifestyle modification including diet and exercise [96]. In genome-wide linkage-scans, a genomic region close to the leptin locus emerged to contribute to the fasting insulin response to exercise training [97]. And in 180 Brazilians, an FTO polymorphism was associated with decreased fasting plasma glucose after 9-month lifestyle intervention [98]. Additionally, polymorphisms in ADIPOR1 [99], PPARG [49], PPARD [100], PPARGC1A (encoding PGC1α) [101], TCF7L2 [102], and SIRT1 [103] were shown to impact the glucose homeostasis response to lifestyle intervention [71].

Exercise also regulates epigenetic modifications [104], in CpG-islands [105], enhancer sites [106, 107], as well as on histones [108]; furthermore, micro-RNA expression changes due to exercise were shown, in plasma [109] and skeletal muscle [110]. There is evidence that different doses of exercise reveal different inflammatory miRNA responses [111]. Notably, insulin sensitivity might influence the epigenetic response to exercise [112]. But investigating the relevance of differences in epigenetic regulation for the variability in exercise response has just started. One study reported highly variable responses in muscle mass upon resistance training and deciphers differentially expressed microRNAs [113].

Impact and interplay of genetic factors for non-response will be specified in the future. Additionally, whether the genetic influence might be overcome by higher training intensities/volumes/types is not clear yet and requires future research.

### Muscle

Skeletal muscle displays one of the most important target tissues of insulin. It accounts for more than 85 % of insulin-dependent glucose uptake [114]; thus, mechanistic studies to elucidate the metabolic adaptation to exercise and its regulation mostly focus on skeletal muscle. The training-induced improvement in glucose disposal has been attributed among other non-muscle adaptations to increases in muscle mass, muscle fiber type switching, mitochondrial biogenesis, and enhanced capillarization [115–117]. On a molecular level, increased abundance and altered posttranslational modifications of proteins important in uptake and oxidation of glucose and fatty acids have been shown [118–121]. Together, enhanced fuel oxidation in muscle appears to be one major key mechanism of improved glucose control after training [24].

Given the relevance of oxidative metabolism in the prevention of poor glucose homeostasis, it was speculated that differences in mitochondrial content and mitochondrial fuel oxidation in response to training might play a role in exercise non-response [43]. In a subgroup of the HART-D study, non-responders were defined as diabetic individuals with constant HbA1c, percent body fat, and BMI, and reduced muscle mitochondria content after exercise [48]. A microarray analysis of muscle biopsies of these non-responders at baseline revealed 186 differentially regulated mRNAs compared with responders, mostly affecting substrate metabolism and mitochondrial biogenesis/function [48]. Increased mRNA levels of genes encoding for mitochondrial proteins were also found in prediabetic responders versus non-responders in terms of glucose tolerance [47]. Higher muscle concentrations of the tricarboxylic acid cycle intermediates were found to correlate best with exercised-induced change in insulin sensitivity [63], at least in a vigorous-intensity exercise group. In 66 untrained participants of a resistance training intervention, a proinflammatory transcript profile was associated with the failure to induce muscle hypertrophy, whereas genes involved in muscle development were uniquely expressed in responders regarding myofiber hypertrophy at baseline [122].

To conclude, the data on specific adaptations in the muscle of responders and non-responders highlight the relevance of mitochondrial pathways for the improvement of metabolic control, independent of different biopsy timings, training regimes, heterogeneous cohorts, and different definitions of metabolic non-response among studies. Notably, for detailed pathomechanisms, we have to differentiate thoroughly between mitochondrial content, OXPHOS capacity, and fat oxidation. An important issue here is to understand the individual variability in these mitochondrial adaptations and the molecular basis for the susceptibility to resist to training intervention.

### Adipose tissue

Adipose tissue contributes relevantly to whole-body metabolism, both as metabolic sink and an endocrine organ [123, 124]. Notably, being obese implies a greater risk for development of type 2 diabetes than being inactive [125]. Improvement of glucose homeostasis after 1 year of combined lifestyle intervention in 104 viscerally obese men was not independently associated with improvement of cardiorespiratory fitness, but with changes in visceral and subcutaneous adipose tissue [46]. Thus, beneficial metabolic improvements seem to be mediated by adipose tissue [46, 72, 126, 127]. That is in line with an observation that there are no weight-independent exercise effects on adipokines [128]. Recent studies in mice affirmed a role for subcutaneous adipose tissue in exercise-induced improvements in glucose homeostasis [129, 130]. On the other hand, anti-inflammatory effects of exercise on adipose tissue are reported to be weight-loss-independent [131].

Effects of exercise affect all fat compartments. General exercise-related changes on adipose tissue comprise fat loss per se, beneficial shifts in body fat composition, altered mitochondrial function, and secretory responses [125, 131–134]. It seems to be established that exercise leads to increased subcutaneous adiponectin mRNA levels, while other adipokines and their systemic relevance are under discussion [132]. In a 6-month supervised exercise intervention in 47 healthy sedentary men [135], genes encoding the respiratory chain, histone subunits, small nucleolar RNAs, ribosomal proteins, and pathways like oxidative phosphorylation were up-regulated, whereas Wnt and mitogen-activated protein kinase (MAPK) signaling pathways were down-regulated due to exercise.

Elevated adipose tissue peroxisome proliferator-activated receptor gamma (PPARg) and PGC1α were early supposed to mediate the beneficial effects of exercise on insulin sensitivity [136]. Also suppressed angiogenesis in white adipose tissue after exercise was associated with insulin resistance [137]. Additionally, endothelial nitric oxide synthase (eNOS) seems to be a major control point in the fragile energy metabolism balance [133], as it gained attention as an inductor of mitochondrial biogenesis [138].

Conversion of white adipocytes to more energy-dissipating brown-like adipocytes is known as browning. This effect might also play a role in adipocytes’ response to exercise [139]. There is further evidence that high physical activity leads to increased brown adipose tissue activity [140]. If browning in humans is of relevant impact, is currently under discussion [141–143]. In this respect, the role of a PGC-1α-dependent exercise-induced myokine and browning factor identified in mice [144], named irisin, was recently very controversially discussed in humans [145–148].

In conclusion, there is good evidence that not only muscle, but also altered adipose tissue metabolism can contribute to non-response.

### Liver

Long-term lifestyle intervention leads to the reduction of intrahepatic lipids [29, 72, 149–151]; this reduction in liver fat mediates a relevant part of the beneficial effects on insulin resistance, more than reduction of other fat compartments does [72]. Furthermore, we and others have shown that liver fat is the most reactive fat compartment in response to a lifestyle intervention [72, 152]. Notably, after 2 h of aerobic exercise, intrahepatic lipids in 18 healthy lean volunteers increased about 35 % from baseline, pointing to intrahepatic lipids as a very flexible fuel store [153] serving as a buffer for excess free fatty acids. Data on molecular alterations in the liver upon exercise are very limited, but exercise studies in mice gave hint for a pronounced regulation of signal transduction and gene expression in the liver [154, 155]. Recent data obtained from liver vein samples verified the hepatic release of FGF21 during exercise in humans [156]. This exercise-dependent regulation of FGF21, a liver-derived factor with possibly beneficial effects on glucose control and body weight regulation [157], opens a further perspective for the individual regulation of exercise response on the level of hepatokines.

### Brain

Exercise enhances functional brain capabilities [158]. Furthermore, exercise was shown to improve whole-body metabolism via the regulation of central control mechanisms: reduced appetite and food intake were reported [159, 160].

Vice versa, high-cerebral insulin sensitivity in humans at baseline was associated with higher loss of body fat during lifestyle intervention [161]. Unfortunately, the cohort was too small to find direct effects on glucose homeostasis, independent of fat loss. Since cerebral insulin sensitivity was found to affect peripheral insulin sensitivity [162, 163] and other brain functions as reviewed in [164], it is conceivable that individual differences of central insulin action are relevant for the response variability to exercise on glucose homeostasis. For further understanding of the exercise-brain-metabolism axis, we will need more human studies.

### Inflammation

A role of subclinical inflammation in the development of obesity and diabetes is widely accepted. This linkage between inflammation and diabetes was extensively shown in various organs, like adipose tissue [165], skeletal muscle [166], and liver [167]. As the issue is very complex, and most of the molecules have both pro- and anti-inflammatory effects, the relevance of exercise-regulated cytokines and chemokines for the prevention or treatment of metabolic diseases is still under debate. Exercise-induced beneficial effects on metabolic control have been linked to several cytokines and chemokines with known functions in inflammatory processes [166]. Additionally, anti-inflammatory influences of regular exercise have been shown in several studies [168, 169]. In brain, anti-inflammatory exercise effects were reported, at least in mice [170]. Thus, although exercise acutely can induce inflammatory processes, predominantly after an unadjusted work load and eccentric exercise [171], it can help to reduce subclinical inflammation in the long run.

For exercise non-response, a role of a differential regulation of pro-/and anti-inflammatory cytokines can only be speculated; recently, this was supposed for skeletal muscle [122].

## Conclusion

**Fig. 1:**
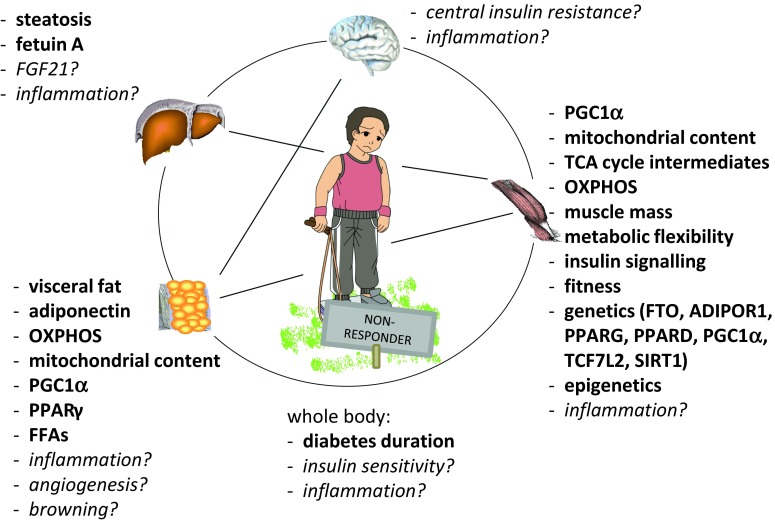
*Hypothetical* and *observed* contribution to exercise non-response with respect to glucose homeostasis. For details, see text

In this review, we discussed individual responses to exercise training in terms of glucose homeostasis; current ideas for underlying pathomechanisms for the lack of improvement in humans were summarized, as illustrated in Fig. 1. In general, we should clearly encourage our patients to increase their physical activity. There are many aspects, e.g., socio-economic, quality of life etc., beyond specific metabolic endpoints, which are worth being an active individual. Nevertheless, personalized adjustments of exercise recommendations are inevitable, different training strategies for individual subgroups may be necessary. Despite the very complex issue (different endpoints, training types, nutrition, populations, highly individual participants, etc.), we hopefully will promote our knowledge to tackle the non-response. Therefore, we do need further studies to unravel detailed mechanisms for insufficient responses to exercise training. Thus, well-designed, supervised studies with an adequate number of participants and clearly defined conditions for non-response are demanded; for elucidation of non-responders’ characteristics and possible pathomechanisms, we should perform pure training interventions in deeply phenotyped cohorts; without noise of additional diet interventions, that should be investigated separately. Novel studies should address the following questions: Who is the non-responder? What are risk factors for non-response? How can we predict the non-response with easy-to-use parameters? Which of several training regimes could overcome non-response? Is interval training the new winner? Is there a correlation of several metabolic endpoints? To what extent? And valid for every individual? What are the underlying molecular pathomechanisms for non-response? Are we able to discriminate discrete pathomechanisms, and what is their impact on whole-body glucose homeostasis?

More detailed, we need exact tissue-specific (using the biopsy technique) measurements, e.g., of mitochondrial content and function, and need to clarify a possible pathogenic role for inflammation. By omics-technology, we could drive new hypotheses, determining further mechanistic studies. Subsequently, this will hopefully generate novel ideas for a potential pharmacological treatment of non-response. Furthermore, with the modern technique of brain imaging available, we should specify the central-nervous influence on exercise non-response. It is mandatory to design supervised studies. There are myriads of large intervention studies showing benefits of exercise; thus, we should concentrate our efforts and funds on the above-mentioned questions. Last but not least, all our results should be feasible for our patients’ daily routine far away from a controlled supervised study setting.
